# First-line support for assistance in breathing in children: statistical and health economic analysis plan for the FIRST-ABC trial

**DOI:** 10.1186/s13063-020-04818-w

**Published:** 2020-10-31

**Authors:** Izabella Orzechowska, M. Zia Sadique, Karen Thomas, Peter Davis, Kevin P. Morris, Paul R. Mouncey, Mark J. Peters, Alvin Richards-Belle, Lyvonne N. Tume, Padmanabhan Ramnarayan, David A. Harrison

**Affiliations:** 1 Clinical Trials Unit, Intensive Care National Audit & Research Centre (ICNARC), Napier House, 24 High Holborn, London, WC1V 6AZ UK; 2 Department of Health Services Research and Policy, London School of Hygiene & Tropical Medicine, 15-17 Tavistock Place, London, WC1H 9SH UK; 3 Paediatric Intensive Care, Bristol Royal Hospital for Children, University Hospitals Bristol NHS Foundation Trust, Upper Maudlin Street, Bristol, BS2 8BJ UK; 4 Paediatric Intensive Care, Birmingham Children’s Hospital, Birmingham Women’s and Children’s NHS Foundation Trust, Steelhouse Lane, Birmingham, B4 6NH UK; 5 Paediatric Intensive Care Unit, Great Ormond Street Hospital for Children NHS Foundation Trust, and NIHR Biomedical Research Centre, London, WC1N 3JH UK; 6 Respiratory Critical Care and Anaesthesia Unit, UCL Great Ormond Street Institute of Child Health, London, WC1N 1EH UK; 7 Children’s Acute Transport Service, Great Ormond Street Hospital for Children NHS Foundation Trust and NIHR Biomedical Research Centre, London, WC1N 3JH UK; 8 School of Health & Society, University of Salford, Mary Seacole Building, Frederick Road Campus, Broad St, Salford, M6 6PU UK; 9 Paediatric Intensive Care Unit, St Mary’s Hospital, Imperial College Healthcare NHS Trust, Praed Street, Paddington, London, W2 1NY UK

**Keywords:** Intensive care, Paediatrics, Respiratory medicine, Statistics, epidemiology and research design, Oxygen delivery

## Abstract

**Background:**

The FIRST-ABC trial comprises of two pragmatic, multicentre, parallel groups, non-inferiority randomised clinical trials designed to evaluate the clinical non-inferiority of first-line use of high flow nasal cannula (HFNC) to continuous positive airway pressure (CPAP) in critically ill children who require non-invasive respiratory support (NRS).

**Objectives:**

To describe the pre-specified statistical and health economic analysis for the FIRST-ABC trial before completion of patient recruitment and data collection.

**Methods:**

The statistical analysis plan was designed by the chief investigators and statisticians. We define the primary and secondary outcomes, summarise methods for data collection and safety monitoring, and present a detailed description of the planned statistical and health economic analysis.

**Results:**

The primary clinical outcome is time to liberation from respiratory support. The primary effect estimate will be the adjusted hazard ratio, reported with a 95% confidence interval. As a sensitivity analysis, the primary analysis will be repeated using time to start weaning of NRS. Subgroup analyses will be performed to test for interactions between the effect of allocated treatment group and pre-specified baseline covariates. The health economic analysis will follow the intention-to-treat principle and report the mean (95% confidence interval) incremental costs, quality-adjusted life years (QALYs) and cost-effectiveness up to 6 months. All analyses will be performed separately for each of the two trials, and any results will not be combined.

**Conclusion:**

The FIRST-ABC trial will assess the non-inferiority of HFNC compared to CPAP in two parallel trials with shared infrastructure (step-up RCT and step-down RCT). We have developed a pre-specified statistical and health economics analysis plan for the FIRST-ABC study before trial completion to minimise analytical bias.

**Trial registration:**

ISRCTN ISRCTN60048867. Registered on 19 June 2019.

## Introduction

### Background and rationale

Increasing recognition of the risks of invasive ventilation for critically ill children, such as ventilator-induced lung injury and nosocomial infections, has prompted greater use of non-invasive respiratory support (NRS) techniques in paediatric intensive care units (PICUs) worldwide [1, 2]. Continuous positive airway pressure (CPAP) is a mode of NRS which is commonly used and effective, but can be uncomfortable for some children, and is associated with a small but significant risk of complications such as air-leak and nasal trauma. An alternate mode of NRS, high-flow nasal cannula (HFNC), has recently gained popularity since it appears easy to use and well tolerated by patients [3–6].

The FIRST-ABC Trial is testing the hypothesis that in critically ill children who require NRS, the first-line use of high flow nasal cannula (HFNC) is non-inferior to CPAP in terms of time to liberation from respiratory support.

This document describes the proposed statistical analysis plan (SAP) for the trial and has been prepared in accordance with published guidelines [7].

### Objectives

To evaluate the clinical and cost-effectiveness of the use of HFNC, as compared with CPAP, when used as the first-line mode of NRS, both as a step-up treatment (*step-up RCT*) and as a step-down treatment (*step-down RCT*), on the time to liberation from respiratory support.

## Study methods

### Trial design and randomisation

FIRST-ABC is a master protocol comprised of two pragmatic, multicentre, parallel group, non-inferiority randomised clinical trials (step-up RCT and step-down RCT) with shared infrastructure. Treatment allocation in each RCT is a 1:1 ratio. Patients are randomised to first-line use of CPAP or HFNC, with randomisation by permuted blocks (with variable block length) stratified by site and age (< 12 months versus ≥ 12 months).

The trials will be run in up to 25 NHS paediatric critical care units (PICUs and/or high dependency units, HDUs) in England, Wales and Scotland.

### Sample size

The sample size was calculated as follows: to achieve 90% power with a one-sided type I error rate of 2.5% to exclude the pre-specified noninferiority margin of HR = 0.75 (corresponding to approximately a 16-h increase in median time to liberation based on pilot RCT data) requires 508 events to be observed. Based on pilot RCT data [8], we also anticipate 5% censoring due to death or transfer, leading to a required sample size of 268 patients per group in each of the two RCTs. To allow for withdrawal/refusal of deferred consent, and for exclusion due to non-adherence in the per protocol (PP) population, we will recruit a total sample size of 600 patients in each of the two RCTs.

### Framework

The primary clinical outcomes will be tested for non-inferiority. Other secondary outcomes will be tested for superiority, where testing is specified, or analysed using descriptive statistics only if no testing is specified in this SAP.

### Statistical interim analysis and stopping criteria

The internal pilot phase will be evaluated 6 months after the first site opened to recruitment against pre-specified progression criteria (number of sites opened, recruitment rate, proportion of patients started on allocated treatment, changes/escalation to other forms of respiratory support and weaning carried out as per protocol).

A single interim analysis will be carried out in each RCT, after recruitment and follow-up to day 60 of 300 patients. At this point, the following endpoints will be analysed in the intention to treat (mITT) population only:

- Time to liberation from respiratory support, which will be tested using an unadjusted log-rank test, with early termination of the trial recommended if any one arm is shown to be superior with *p* < 0.001 (Peto-Haybittle stopping rule)
- Mortality to day 60, which will be tested using a log-rank test, with early termination of the trial recommended if any one arm is shown to be superior with *p* < 0.05.

For this interim analysis, patients discharged alive from hospital with no further death after discharge recorded are assumed to be alive on the day of data extract. Patients who have withdrawn or refused consent for access to medical records will be censored on the date of withdrawal or refusal of consent.

### Timing of final analysis

The final analysis for each RCT will be performed no earlier than 6 months after the last patient has been randomised to that RCT.

### Timing of outcome assessments

Following randomisation, details of respiratory support are recorded hourly for the first 6 h, and six hourly thereafter until the end of respiratory support (or to at least 48 h following randomisation, if patients are transferred to another unit or ward in a different hospital). At the relevant timepoints, physiological parameters (e.g. fraction of inspired oxygen (Fi0_2)_ Oxygen saturation (Sp0_2_)) and sedation use are recorded if patients are on either HFNC or CPAP. Patient comfort is assessed at least six hourly while patients are on HFNC or CPAP.

Survival status is recorded at unit discharge, at ultimate discharge from critical care (if the patient has been transferred to another critical care unit), at discharge from acute hospital and at days 60 and 180 post-randomisation.

Parental stress is measured using the Parental Stressor Scale: PICU [9] at/around the time of consent (anticipated to be within 24–48 h of randomisation). Quality of life (measured using age-appropriate Pediatric Quality of Life Inventory (Peds-QL) [10] and The Child Health Utility 9D (CHU-9D) [11]) and health services/resource use is assessed at 6 months post-randomisation.

## Statistical principles

### Confidence intervals and *p* values

The primary clinical outcome will be tested for non-inferiority. Other secondary outcomes will be tested for superiority, where testing is specified, or analysed using descriptive statistics. Statistical tests will be two-sided with significance set at *P* < 0.05 unless otherwise specified. Effect estimates will be reported with 95% confidence intervals. There will be no adjustment for multiple testing. The results of subgroup analyses will be interpreted taking into account accepted criteria for credible subgroup effects [12, 13]. All analyses described in this SAP will be performed separately for each of the two RCTs, and any results will not be combined.

### Adherence and protocol deviations

#### Exposure

Exposure to the intervention will be assessed by the following parameters, which will be calculated for each treatment group and summarised using descriptive statistics (mean, standard deviation, median and interquartile range (IQR), or counts and percentages for binary and categorical variables) unless otherwise specified:

- In patients randomised to CPAP, pressure (in cm H_2_O as a continuous variable, and grouped as < 7 cm, 7–8 cm, > 8 cm), by hour during the first 6 h from randomisation
- In patients randomised to HNFC, flow rate (as % of recommended starting rate, and group ed. as ≤ 50%, 51–75%, 76–85%, 86–95%, ≥  95% of recommended starting rate), by hour during the first 6 h from randomisation
- Time from first recorded observation meeting weaning/failure/stopping criteria to time of weaning/switch or escalation/treatment stop

Further treatment patterns across each group and time from first meeting weaning criteria to start of weaning attempt will be explored using summary statistics and graphic methods only; no formal statistical testing will be performed.

#### Protocol adherence

The number and % of patients will be reported for each of the following potential protocol deviations: did not start randomised treatment (with details provided on what respiratory support, if any, was started), switched or escalated respiratory support without meeting treatment failure criteria; weaning attempt made without meeting weaning criteria, respiratory support is discontinued while FiO_2_ ≥ 0.3 and moderate or severe respiratory distress is documented.

### Analysis population

All randomised patients will be included in the intention-to-treat (ITT) population. A modified ITT (mITT) population will be used for analysis of the primary endpoint, consisting of the ITT populations excluding those with no recorded respiratory support post-randomisation. The per protocol (PP) population will consist of all randomised patients who met the eligibility criteria and were started on the randomised respiratory support, as the first respiratory support post-randomisation.

## Trial population

### Screening data

The following summaries of screening will be presented for each trial:

- Number and % of patients screened who did not meet inclusion criteria, overall and by criteria
- Of the patients who met the inclusion criteria, number and % who met exclusion criteria, overall and by criteria
- Of the eligible patients (i.e. met inclusion criteria and did not meet exclusion criteria), number and % not randomised, overall and by reason (if known)

### Eligibility

#### Inclusion criteria

1. Admitted/accepted for admission to PICU/HDU
2. Age > 36 weeks corrected gestational age and < 16 years
3. Assessed by the treating clinician to require NRS, *EITHER*
  A. For an acute illness (step-up RCT) *OR*
  B. Within 72 h of extubation following a period of invasive ventilation (step-down RCT)

#### Exclusion criteria

1. Assessed by the treating clinician to require immediate intubation and invasive ventilation due to severe hypoxia, acidosis and/or respiratory distress, upper airway obstruction, inability to manage airway secretions or recurrent apnoeas
2. Tracheostomy in place
3. Received HFNC/CPAP for > 2 h in the prior 24 h
4. On home non-invasive ventilation prior to PICU/HDU admission
5. Presence of untreated air-leak (pneumothorax/pneumomediastinum)
6. Midfacial/craniofacial anomalies (unrepaired cleft palate, choanal atresia) or recent craniofacial surgery
7. Agreed ‘not for intubation’ or other limitation of critical care treatment plan in place
8. Previously recruited to the FIRST-ABC trial*
9. Clinician decision to start other form of NRS (i.e. not HFNC or CPAP)

*i.e. patients randomised to the step-up RCT will not be eligible for randomisation to the step-down RCT. Similarly, patients once enrolled to the step-up or step-down RCTs and satisfied the primary outcome of being liberated from respiratory support will not be eligible for re-randomisation to the trial even if they require further episode(s) of NRS during the same or on subsequent hospital admissions.

Full details of the study population including eligibility, randomisation and treatment delivery can be found in the FIRST-ABC trial protocol [14].

### Recruitment

CONSORT [15] flow diagrams for the ITT and PP populations will be completed for each trial. These will include number and percentage of patients starting respiratory support post-randomisation, and numbers and percentage of patients by availability of primary endpoints.

### Consent and withdrawal

The parent/legal guardian of trial participants will be asked to consent to one or more aspects of the trial (continued participation/treatment; ongoing data collection; completion of the Parental Stressor Scale: PICU, to receive a follow-up questionnaire at 6 months post-randomisation; sharing of anonymised data) as soon appropriate and practical after randomisation (usually within 24–48 h of randomisation). Once given, consent can be withdrawn at any time up to the end of the study. Data collected up to the point of refusal or withdrawal of consent to data collection will be retained, unless parents specifically request otherwise.

If a patient is transferred to another hospital while still on trial treatment, all efforts will be made to continue to collect a minimum trial dataset (including the primary outcome measure).

### Baseline patient characteristics

Baseline data is collected at critical care admission via data linkage to Paediatric Intensive Care Audit Network (PICANet), and directly via trial CRF for physiology at randomisation. The following baseline demographic and clinical data will be summarised in the mITT and PP populations, by allocated treatment group (using mean, standard deviation, median and interquartile range (IQR), or counts and percentages for binary and categorical variables), but not subjected to statistical testing:

In both trials: age (continuous in years, and by age group ≤ 28 days, 29–180 days, 181–364 days, 1–4 years, 5–10 years, 11–15 years), sex; respiratory distress (mild, moderate or severe), heart rate (bpm and in age-corrected centiles), SpO2, FiO2, SpO2/FiO2 ratio, COMFORT-B score [16] (where available), presence of pre-specified comorbidities, reason for admission (step-up RCT) or reason for invasive ventilation (step-down RCT).

In the step-up trial only: respiratory support received in 24 h prior to randomisation (type and duration), whether on respiratory support at time of randomisation, general anaesthesia for surgery/procedure in the 6 h preceding randomisation.

In the step-down trial only: duration of invasive ventilation (continuous in hours, and by duration < 5 days vs. ≥ 5 days).

## Analysis

### Outcome definitions

#### Primary outcome

- The primary clinical outcome is time to liberation from respiratory support, defined as the time from randomisation to the start of a 48-h period during which the child was free of all forms of respiratory support.

#### Secondary outcomes

- *Mortality at PICU/HDU discharge, day 60 and day 180*. Mortality at discharge from the PICU/HDU will be defined as death due to any cause before discharge to any location providing a level of care less than level 2 (high dependency care). Mortality at day 60 and 180 will be calculated as binary endpoints using all patients with known survival status at those times, and additionally using time to event methods with surviving patients censored at date last known to be alive (to a maximum of day 180).
- *Rate of (re) intubation at 48 h*. Intubation at 48 h is defined as present if the child has started any invasive ventilation at any time up to and including 48 h and zero minutes after time of randomisation. Patients are included in the denominator if they have received invasive ventilation by 48 h, or are known not to have received any invasive ventilation from randomisation to 48 h following randomisation. Patients discharged from PICU/HDU before 48 h are assumed not to have been invasively ventilated post discharge.
- *Duration of PICU/HDU and acute hospital stay*. Duration of PICU/HDU will be calculated as the sum of the duration (in days and fractions of days) from the date and time of randomisation to the date and time of first discharge from a critical care unit (or ultimate discharge from critical care if transferred directly to another critical care unit) or to death in the critical care unit. Duration of acute hospital stay will be calculated as the duration in days from the date of randomisation to the date of ultimate acute hospital discharge or death in acute hospital.
- *Patient comfort, during randomised treatment and during NRS (i.e. HFNC and/or CPAP), measured using the COMFORT-B score*. Patient comfort is measured during HFNC or CPAP using the COMFORT-B score which will be summarised at patient level using the median of all recorded scores. To be measured in all patients with at least one recorded COMFORT-B score in the first 6 h of support following randomisation, AND, while respiratory support continues, at least one COMFORT-B score per day during at least the first 48 h of respiratory support.
- *Proportion of patients in whom sedation is used during NRS*. Need for sedation will be defined as the proportion of patients in whom sedation is used NRS at any point until liberation from respiratory support. Patients will be included in the denominator if they have a minimum of three non-missing observations in the first 6 h of respiratory support, AND, while respiratory support continues, at least two non-missing observations per day during the first 48 h of respiratory support.
- *Parental stress, in hospital at/around the time of consent, measured using the Parental Stressor Scale: PICU.* Parental stress will be measured using the validated Parental Stressor Scale: PICU (PSS: PICU) in hospital at/around the time of consent (anticipated to be within 24–48 h post-randomisation). This consists of 37 items each scored in whole numbers from 1 (not stressful) to 5 (extremely stressful). A total score is calculated as the mean of all completed items [9].
- *Health-related quality of life at 6 months.* Health-related quality of life at 6 months will be measured using the age-appropriate Paediatric Quality of Life Inventory (Peds-QL)32 and the Child Health Utility 9D (CHU-9D) completed by parents at 6 months post-randomisation. The PEDS-QL instrument uses a different set of question of each age group of 1–12 months, 13–24 months, 2–4 years, 5–7 years, 8–12 years and 13+ years. For each age group, an overall score is calculated on a scale of 0–100 with higher scores indicating better quality of life. In infants under 2 years, five subscales are defined, relating to physical functioning, physical symptoms, emotional functioning, social functioning and cognitive functioning, and in children of 2 years and over, four subscales are defined, relating to physical functioning, emotional functioning, social functioning and school functioning [10]. CHU-9D was developed with children aged 7–17 and is designed to produce utility values for use in calculating quality-adjusted life years (QALYs) [11].
- *Total costs at 6 months.* Cost will be calculated from patient-level resource use data on resources required to deliver the intervention, length of stay in PICU/HDU and acute hospital, for the index admission and any readmissions before 6 months, and use of personal health services after acute hospital discharge within 6 months post-randomisation. Patient-level resource use data will be valued using appropriate unit costs data from the NHS Payment by Results database, unit costs of health and social care (PSSRU) and from local Trust Finance Departments, to calculate total costs at 6 months.
- *Quality-Adjusted Life Years (QALYs) at 6 months*. The health outcome for the economic evaluation will be summarised using QALYs, which unites quantity (survival) and quality of life into a single metric. To do this, HRQoL, which is measured on an index scale of 1 (equals full health) and 0 (equals death), at 6 months will be assessed using the CHU-9D instrument, with valuation using the validated UK tariffs [11]. HrQoL data will be combined with the survival data to calculate QALYs at 6 months. QALYs will be calculated by valuing each patient’s survival time by their HrQoL at 6 months according to the “area under the curve” approach. For 6-month survivors, QALYs will be calculated using the CHU-9D scores at 6 months, assuming an CHU-9D score of zero at randomisation, and a linear interpolation between randomisation and 6 months. For decedents between randomisation and 6 months, we will assume zero QALYs.
- *Incremental net monetary benefit gained at a willingness-to-pay of £20,000 per QALY at 6 months associated with HFNC vs. CPAP.* Net monetary benefits will be calculated by valuing QALY gains at the NICE recommended (£20,000) willingness to pay (WTP) for a QALY gain and subtracting incremental costs.

### Clinical effectiveness analysis methods

#### Primary outcome

The median (IQR) time to liberation from respiratory support will be reported for each arm using Kaplan-Meier estimates and compared between groups using Cox regression, unadjusted and adjusted for important baseline characteristics (including shared frailty at the site level). The covariates for inclusion in the regression models are the following, which have been selected a priori based on an established relationship with outcome for critically ill children:

In both RCTs:

- Age (< 12 months versus ≥ 12 months)
- Severity of respiratory distress at randomisation (severe versus mild/moderate)
- SpO2:FiO2 ratio at randomisation (linear)
- Co-morbidities (none versus neurological/neuromuscular versus other)

Step-up RCT only:

- Reason for admission (bronchiolitis versus other respiratory (airway problem, asthma/wheeze or any other respiratory) versus cardiac versus other (neurological, sepsis/infection, any other)
- Whether the patient was on NRS at randomisation (yes/no)

Step-down RCT only:

- Length of prior invasive mechanical ventilation (IMV) (< 5 days versus ≥ 5 days)
- Reason for IMV (cardiac versus other)

The primary effect estimate will be the adjusted hazard ratio, reported with a 95% confidence interval. HFNC will be considered non-inferior to CPAP if the lower bound of the 95% confidence interval is above 0.75 in both the mITT and PP populations. Patients without a recorded time of liberation will be censored at date and time of death (for patients who died while on treatment) or at date and time of last recorded respiratory support. The assumption of proportional hazards will be explored by fitting a Cox model with time-dependent covariates.

Exploratory analyses will test for an interaction between the subgroup categories defined below (or subgroup variable for linear interactions) and the treatment group in a multilevel Cox regression model, adjusted for the same baseline variables as the primary analysis. For linear interactions, the interaction effect will be illustrated by calculating the adjusted hazard ratio within five categories at quintiles of the continuous variable. Each subgroup will be considered separately:

In both RCTs:

- Age
- Severity of respiratory distress at randomisation
- SF ratio at randomisation (interaction test as a linear covariate, with effect illustrated by calculating the adjusted hazard ratio within five categories at quintiles of the continuous variable)
- Co-morbidities

Step-up RCT only:

- Reason for admission
- Whether the patient was on NRS at randomisation

Step-down RCT only:

- Length of prior IMV
- Reason for respiratory support post-extubation, categorised as planned (randomisation followed by extubation), indeterminate (extubation followed by randomisation within 60 min of extubation) vs rescue (extubation followed by randomisation more than 60 min post extubation) breathing support
- Reason for IMV

We will treat age as a continuous variable and determine whether the model goodness-of-fit is better versus treating age as a categorical term for any analyses focusing on those over the age of 12 months. We anticipate a high proportion of patients will be aged < 12 months, and therefore, exploration of age effects in the older ages will only be conducted if there are sufficient patient numbers.

As a sensitivity analysis, the primary analysis will be repeated using time to start weaning of NRS (i.e. duration of ‘acute’ respiratory support), time to meeting objective ‘readiness to wean NRS’ criteria and time from start of support to liberation from support.

#### Secondary outcomes

Binary outcomes (mortality at discharge from critical care, at 60 and 180 days post-randomisation, (re) intubation at 48 h, sedation use during randomised treatment, sedation use during HFNC or CPAP) will be reported in each treatment group, in the PP and mITT populations. Absolute risk reduction and unadjusted odds ratios will be reported with 95% confidence intervals. Multilevel logistic regression (adjusted for the same baseline variables as the adjusted analysis of the primary outcome) will be used to calculate adjusted odds ratios with 95% confidence intervals.

Continuous outcomes (duration of PICU/HDU and hospital stays) will be summarised by treatment groups, stratified by survival status, in the PP and mITT populations. Mean difference between groups will be calculated, with 95% confidence interval using bootstrapping to account for anticipated non-normality in the distribution.

Duration of survival to day 180 will be plotted as Kaplan-Meier survival curves, in the PP and mITT populations, and unadjusted and adjusted hazard ratios with 95% confidence intervals will be calculated using Cox regression models.

Parent-reported outcomes (PSS: PICU score, PEDS-QL score, CHU-9D score) will be summarised by treatment groups, in the PP and mITT populations. Mean difference between groups will be calculated, with 95% confidence interval using bootstrapping to account for anticipated non-normality in the distribution. Linear regression will be used to calculate adjusted mean differences.

For each patient, their median Comfort-B score while on randomised treatment and their median COMFORT-B score while on either HFNC or CPAP will be calculated. These median scores will be summarised by treatment groups, using median (IQR) and mean (sd). The number and % of patients with any recorded COMFORT-B score ≥ 23 while on randomised treatment and the number and % of patients with any recorded COMFORT-B score ≥ 23 while on either HFNC or CPAP will be reported. Mean difference between groups will be calculated, with 95% confidence interval using bootstrapping to account for anticipated non-normality in the distribution. Linear regression will be used to calculate adjusted mean differences.

### Cost-effectiveness analysis methods

A full cost-effectiveness analysis (CEA) will be undertaken to assess the relative cost-effectiveness of HFNC versus CPAP according to the intention-to-treat principle. Resource use and outcome data collected as a part of the trial will be used to report cost-effectiveness at 6 months by randomised treatment group.

Following recent National Institute for Health and Care Excellence (NICE) recommendations, the cost-effectiveness analysis will take a health and personal health services perspective. The primary sources of the resource use data will be the FIRST-ABC trial case report forms (CRFs), PICANET data, hospital episode statistics (HES) database and individual health service questionnaires (HSQ) on the use of personal health services which are posted to surviving patients at 6 months following randomisation. Resource use data from the PICU/HDU stay will be taken from the CRF and linked to routine data from PICANet. Data on the level of care for PICU/HDU bed-days will be gathered through routine collection of the Paediatric Critical Care Minimum Dataset (PCCMDS) in the participating centres via the PICANet database. Information on subsequent PICU/HDU and hospital admissions will be obtained via data linkage with PICANet and HES database. Use of primary care and community health services will be assessed by HSQ at 6 months. Resource use data from the trial datasets, PICANet data, HES database and 6 months’ follow-up questionnaires will be combined with unit costs from the NHS Payment by Results database, unit costs of health and social care (PSSRU) and from local Trust Finance Departments, to report the total costs per patient at 6 months for both randomised groups.

Missing data in costs and HrQoL will be handled with multiple imputation, assuming the data are missing at random (MAR) conditional on the observed data (see below for details on methods used to handle missing data). On the imputed datasets, the cost-effectiveness analysis will use a Bivariate Seemingly Unrelated Regression model to allow for correlation between costs and QALYs and multilevel structure of the data. We will calculate the interclass correlation coefficient (ICC) which measures the proportion of the overall variation that occurs at the cluster level [17]. If ICC > 10%, we will use multilevel models (MLM) to handle clustering and avoid potential biases and incorrect inferences. The incremental results from multiply-imputed datasets will summarised using Rubin’s rule [18].

The CEA will follow the intention-to-treat principle and report the mean (95% confidence interval) incremental costs, QALYs and net monetary benefit at 6 months. The base case analysis will report the incremental effects of randomisation to a HFNC strategy versus CPAP. We will also report the probability that the intervention compared to usual care is cost-effective at different levels of WTP for a QALY gain.

#### Sensitivity analysis for cost-effectiveness

The following sensitivity analyses will be performed to check the robustness of primary CEA results at 6 months.

- *HRQoL data:* A mapping technique will be used to predict the CHU-9D scores from the PedsQL responses [19]. We will also explore alternative distributional assumptions for QALYs.
- *Cost data:* Because of the likely skewed distribution of costs, we will consider several distributions that can give a better fit of cost data. We will assess the implications of potential double-counting of inpatient costs (e.g. costs for vasopressors) across the three sources of resource data.

## Handling of missing data

As the primary endpoint will be analysed using time-to-event methods, patients with missing data will be included in the analysis as censored at the point of last recorded NRS. Time to censoring will be compared between arms using Kaplan-Meier curves to explore the assumption of censoring at random, and if necessary, a competing risk approach may be taken with death and/or withdrawal of consent while on treatment considered as a competing risk.

Multiple imputation will be used to complete missing data in secondary outcomes, costs and HRQoL, under the assumption that the responses are missing at random (MAR) conditional on the observed data. Multiple imputation will be undertaken using the Multivariate Imputation using Chained Equations algorithm, with the model including all baseline variables included in the adjusted models and all outcome variables. The number of imputations will be determined according to level of missingness in the outcome variables. Models will be fitted in each imputed dataset and results combined using Rubin’s rules.

## Additional analyses

The primary analysis will be repeated adjusting for adherence to allocated intervention using a structural mean model with an instrumental variable of allocated treatment to estimate the complier average causal effect of treatment. Adherence will be measured for each patient as the proportion of all events (weaning, escalation, switch or withdrawal of support) which were classified as non-adherent, where for each observation non-adherence is as previously defined under the section headed 'Protocol adherence'. Children who did not start on the randomised treatment will be recorded as having 100% non-adherence. A descriptive analysis of baseline characteristics and some secondary outcomes (mortality and length of stay outcomes, where available) will be performed for patients who did not start any respiratory support post randomisation (i.e. those excluded from the mITT analysis).

## Safety

Specified adverse events (nasal trauma, facial/neck trauma, abdominal distension, pneumothorax, pneumomediastinum, subcutaneous emphysema, facial thermal injury, respiratory arrest, cardiac arrest), and any other possibly related adverse event, are recorded from randomisation up to 48 h after date/time of liberation of respiratory support.

The percentage of patients experiencing one or more adverse event in patients who commenced respiratory support post randomisation will be compared between groups using Fisher’s exact test. Counts and percentages of adverse events, and serious adverse events, overall and by type, will be presented by allocated treatment group.

## Statistical software

All analyses will be conducted in Stata/SE Version 16.1 64-bit x86-64 (StatCorp LLC, College Station, TX). Some additional cost-effectiveness analysis may be carried out in R if required.

## Data Availability

The datasets generated and/or analysed during the current study are not publicly available but are available from the corresponding author on reasonable request.
